# Babesiosis in the immunocompromised population: Results from a multicentric cohort study conducted in Italy

**DOI:** 10.1016/j.parepi.2024.e00372

**Published:** 2024-07-30

**Authors:** Anna Barbiero, Simona Gabrielli, Lapo Dani, Michele Spinicci, Filippo Lagi, Gregorio Basile, Francesca Nacci, Antonia Mantella, Seble Tekle Kiros, Angela Pieri, Andrea Delama, Chiara Piubelli, Salvatore Scarso, Andrea Angheben, Marcello Feasi, Bianca Granozzi, Giorgia Comai, Stefania Varani, Lorenzo Zammarchi, Alessandro Bartoloni

**Affiliations:** a Department of Clinical and Experimental Medicine, Università degli Studi di Firenze, 50134 Firenze, Italy; b Department of Public Health and Infectious Diseases, Sapienza Università di Roma, 00185 Roma, Italy; c Department of Infectious and Tropical Diseases, Azienda Ospedaliero Universitaria Careggi, 50134 Firenze, Italy; d Regional Referral Center for Tropical Diseases, Azienda Ospedaliero Universitaria Careggi, 50134 Firenze, Italy; e Section of Rheumatology, Department of Clinical and Experimental Medicine, Università degli Studi di Firenze, 50125 Florence, Italy; f Department of Infectious Diseases, Provincial Hospital of Bolzano (SABES-ASDAA), Bolzano-Bozen, Italy; g Lehrkrankenhaus der Paracelsus Medizinischen Privatuniversität, Bolzano, Italy; h Infectious Diseases Unit, Trento Hospital, 38122 Trento, Italy; i Department of Infectious, Tropical Diseases and Microbiology, IRCCS Sacro Cuore-Don Calabria Hospital, 37024 Negrar di Valpolicella, (VR), Italy; j Department of Infectious Diseases, Galliera Hospital, 16128 Genova, Italy; k Infectious Diseases Unit, IRCCS Azienda Ospedaliero-Universitaria di Bologna, 40138 Bologna, Italy; l Nephrology, Dialysis and Renal Transplant Unit, IRCCS Azienda Ospedaliero-Universitaria di Bologna, 40138 Bologna, Italy; m Unit of Microbiology, IRCCS Azienda Ospedaliero-Universitaria di Bologna, 40138 Bologna, Italy; n Department of Medical and Surgical Sciences, University of Bologna, 40138 Bologna, Italy

**Keywords:** Babesia, Tick-borne diseases, Italy, Immunosuppression, HIV, Ticks

## Abstract

Human babesiosis is an emerging zoonotic disease; diffused especially in some regions of the United States, it has been less frequently observed in other continents, including Europe. Serological surveys suggest that babesiosis could be more frequent than expected in European countries, representing an emerging health-issue and a possible harm, especially in immunocompromised populations. Only one case of human babesiosis has been reported in Italy and data about the diffusion of the pathogen in this country are scant. We conducted a multicentric serological survey in 5 centers of North-Eastern Italy, aimed to detect the seroprevalence of *Babesia* spp. antibodies in 3 groups of immunocompromised patients: people living with HIV (PLHIV), rheumatologic patients undergoing immunosuppressive therapies and patients undergoing renal transplant. Among the 433 enrolled patients, 3 (0.7%) tested positive for *Babesia* spp. serology. All positive patients belonged to the PLHIV group, with a seroprevalence of 1.7% (3/180) in this population; the three serologically positive patients were all asymptomatic. They were all enrolled in the provinces of Bolzano and Trento, where seroprevalences of 3.1% and 3.6% were recorded, respectively. Our results suggest that further research is needed on this field, awareness should be raised toward the human disease in Europe, especially in immunocompromised patients, and this emerging health issue should be analyzed in a One-Health perspective to be fully understood.

## 1 Introduction

Babesiosis is an emerging tick-borne zoonosis caused by pathogens of the genus *Babesia* spp., an obligated intracellular haematic protozoan that belongs to the phylum of Apicomplexa. *Babesia* was firstly identified by Victor Babes in 1888 in Romania, while studying the cause of a seasonal epidemic “febrile haemoglobinuria” among cattle (Babes, 1888). >100 *Babesia* species are able to infect many vertebrate hosts across the globe; the three main species in Europe for which zoonotic potential has been identified are *Babesia divergens*, *Babesia venatorum* and *Babesia microti*; their main hosts are cattle, roe deer (*Capreolus capreolus*) and small mammals, respectively (Häselbarth et al., 2007; Herwaldt et al., 2003; Yang et al., 2021). In Europe, the first reported case of human babesiosis was described in 1956 in Croatia, involving a splenectomised farmer. The infection was caused by *B. divergens* and the case ended fatally (Skrabalo and Deanovic, 1957). Subsequently, many cases of babesiosis have been reported sporadically in Europe, being caused mostly by *B. divergens* and transmitted to humans through *Ixodes ricinus* tick bites (Zintl et al., 2003). On the other hand, *B. microti* has been reported as the principal etiologic agent of human babesiosis in the U.S.A., where its principal vector is represented by *Ixodes scapularis (*Homer et al., *2000**)*. Nowadays, babesiosis is endemic in this area (especially in the Northern Midwest and the North-East), while it is present to a much lesser extent in Europe (Westblade et al., 2017). Indeed, around 60 cases of human babesiosis have been reported in Europe, with approximately 40 of them attributed to *B. divergens* and a minority of cases attributed to *B. venatorum* and *B. microti*. Most cases have been recorded in areas where livestock is strongly present and where babesiosis also constitutes a significant cause of loss for cattle industry (Zintl et al., 2003; Purnell, 1981). The relatively low number of cases reported in Europe is in contrast with the significant seroprevalence rates that have been detected in several European areas. This could suggest, on one hand, the possibility of frequent asymptomatic infections, but on the other hand, diffused lack of awareness and diagnostic tools, which can lead to misdiagnosis and underreporting (Azagi et al., 2021; Gorenflot et al., 1998; Gabrielli et al., 2014; Lempereur et al., 2015; Foppa et al., 2002; Hunfeld et al., 2002).

The severity of the infection in humans ranges from asymptomatic forms to rapidly fatal ones. The main risk factors for symptomatic and/or severe forms are immunosuppression and especially asplenia. However, moderate and severe clinical presentations have been described in the case of *B. microti* infection also in normosplenic and non-immunocompromised patients (Hildebrandt et al., 2021). Most common complications of the disease are haemolysis, acute respiratory distress and multiorgan failure, leading to death in some cases (Hunfeld et al., 2008).

In Italy, although diffusion of *Babesia* spp. has been documented in several animal species, babesiosis has been rarely reported in humans. Although the first ever documented case of *B. venatorum* infection in humans was detected in this country in 2004, no other cases have been reported afterwards, neither caused by *B. venatorum* or other *Babesia* species. On the other hand, a serosurvey conducted in the North-Eastern regions of Italy showed high percentages of antibody positivity against *Babesia* species in exposed subjects (Gabrielli et al., 2014), suggesting a higher diffusion of this protozoan infection than previously believed.

The lack of data and reports from the Italian country is likely due to underdiagnosis and underreporting, but probably also to the characteristics of babesiosis, which presents frequently with mild and non-specific symptoms. In addition, diagnostic tests, such as PCR and serologic analysis, are not routinely performed and require a reference laboratory. These factors could contribute to a silent spread of babesiosis through our country, therefore suggesting the need of improving our knowledge about the epidemiology of this protozoan infection. Moreover, with the objective of a more profound comprehension of the ecological mechanisms, which constitute the base of *Babesia* spp. spread, a “One-Health” approach is needed in order to get more information about how climate change, deforestation, change in human activities and human habits, are influencing the dynamics of this zoonotic disease.

Finally, if much is known on the clinical course of symptomatic babesiosis in splenectomised patients, literature is scarce when it comes to clinical manifestations of babesiosis in normosplenic, immunodepressed subjects, such as patients treated for rheumatologic disorders, people living with HIV (PLHIV) and solid organ transplant patients, which represent a constantly growing group of the population (Pawełczyk et al., 2019; Perdrizet et al., 2000; Mascarenhas et al., 2018; Benezra et al., 1987; Froberg et al., 2004; Ather et al., 2017; Vyas et al., 2007).

As part of the multicentric study “Emerging blood protozoa in the immunocompromised patient: new strategies for screening, diagnosis, monitoring and clinical management (PROEMA)”, aimed at identifying effective methods for screening and monitoring infections by *Leishmania* spp., *Trypanosoma cruzi* and *Babesia* spp. in immunocompromised patients, we conducted a seroprevalence and molecular survey aimed at establishing distribution of babesiosis among PLHIV, rheumatologic patients and renal transplant recipients in different regions of Central and Northern Italy.

## 2 Materials and methods

### 2.1 Study design and patients' enrolment

A multicentric prospective study, coordinated by the Microbiology Unit of Bologna University Hospital, was conducted between March 2018 and April 2023. Patients were recruited by the following clinical centers: the department of Infectious, Tropical Diseases and Microbiology of Sacro Cuore Don Calabria Hospital in Negrar, Verona, the department of Infectious and Tropical Diseases of Careggi University Hospital in Florence, the department of Infectious Diseases of Galliera Hospital, in Genova, the department of Infectious Diseases of Bolzano Hospital, the department of Infectious Diseases of Trento Hospital, the Nephrology, Dialysis and Renal Transplant Unit and the Infectious Disease Unit of Bologna University Hospital.

Patients were enrolled when presenting for routine control blood exams, with the following inclusion criteria:

- age ≥ 18 years;
- living in Italy for at least two years before enrolment.

In addition, one of the following was also required:

- diagnosis of HIV infection from less than one year;
- HIV infection with CD4+ T-lymphocytes count <350 cells/mm3;
- receiving a kidney graft. Patients were enrolled in the study at the time of transplant;
- ongoing immunosuppressive therapy due to rheumatological or immune-mediated diseases.

After May 2021, another inclusion criterion was added, that is history of tick bite and/or history of any tick-borne disease in the past; in the clinical centres of Trento and Bolzano, patients reporting history of outdoor activities, even without clear history of previous tick bites or tick-borne diseases, were also enrolled. This was done in order to enrol patients with higher possibilities of previous contact with *Babesia* parasite. Since ticks are much more present in Trento and Bolzano, and tick bites are common in people living in this area but often pass undetected, enrolling criteria were less stringent for this specific area.

At the time of enrolment, patients completed a questionnaire, which gathered information about main demographic and anamnestic clinical features, presence of suggestive symptoms for babesiosis, risk factors such as history of tick bite or tick-borne diseases, splenectomy, blood transfusions, frequent outdoor activities.

### 2.2 Screening methods for *Babesia* spp. infection

Blood samples were obtained from enrolled patients in order to perform:

- *in-house* serological assays on serum samples by using *B. divergens* antigens from in vitro culture, as previously described (Gabrielli et al., 2012);
- conventional and quantitative (q)PCRs on EDTA whole blood samples targeting a 800 bp and 120 bp fragment, respectively, of the 18S rRNA gene from zoonotic *Babesia* species, as previously described (Gabrielli et al., 2016a).

### 2.3 Ethical considerations

Ethical approval was given by the local committees of all recruiting centres (PROEMA_2018, study n. 144/2018/Sper/AOUBo, approved on 18/04/2018 for the Microbiology Unit of Bologna University Hospital) all patients signed a written informed consent, data were anonymized, and Good Clinical Practice recommendations were followed. The study was performed in accordance with Helsinki declaration.

The database will be available on request addressed to the corresponding author.

## 3 Results

Between March 2018 and April 2023, a total of 433 patients underwent screening for *Babesia* spp. infection.

The median age of our population was 51 years; 139 (32.1%) patients were females and 294 (67.9%) were males. Among the 433 recruited patients, 180 (41.6%) belonged to the PLHIV group, 123 (28.4%) were undergoing renal transplant and 130 (30.0%) were patients undergoing an immunosuppressive therapy.

**Fig. 1:**
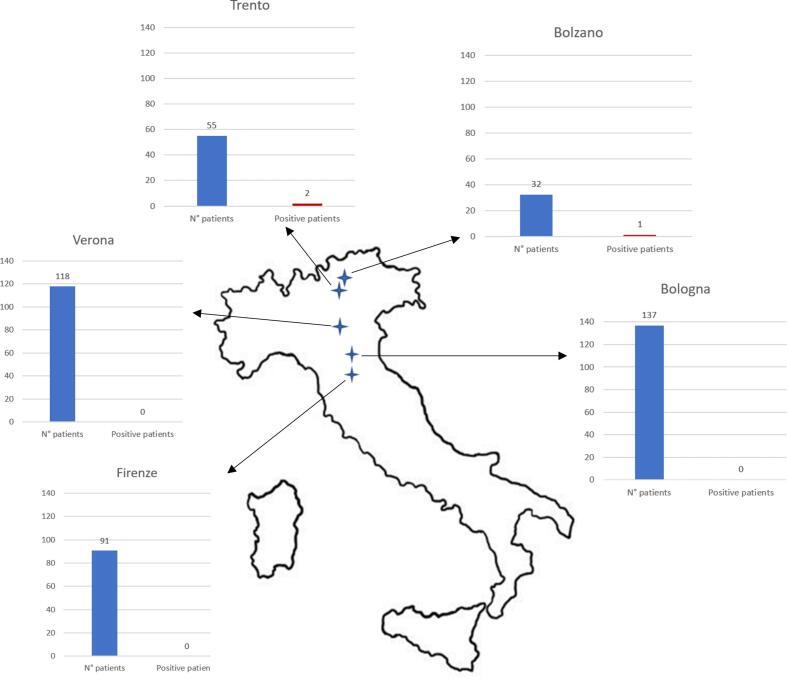
Distribution of recruited patients and positive serological tests divided by area of enrollment.

As regarding those patients for whom information was available, 61/338 (18.0%) had a history of tick bite and no one reported history of tick-borne diseases; 2/179 (1.1%) had history of splenectomy and 61/332 (18.4%) had history of haemo-derivate transfusion; 63/345 (18.3%) reported history of travelling outside Italy in the year before being enrolled and 179/340 (52.6%) reported history of frequent outdoor activities. Among tested population, 87/433 (20.1%) were enrolled in mountainous areas of North-Eastern Italy, particularly in the areas of Trento province (55/433, 12.7%) and Bolzano province (32/433, 9.7%); 92/433 (21.3%) were enrolled in Tuscany region, 118/433 (27.3%) in Veneto region, 136/433 (31.4%) in Emilia-Romagna region (Fig. 1). Three out of 433 (0.7%) patients tested positive at the serology for *Babesia* (Table 1). When considering only those patients who lived in the provinces of Trento or Bolzano 3.6% (2/55) and 3.1% (1/32) of screened patients tested positive for *Babesia* serological tests, respectively (Fig. 1). All the positive screening results belonged to the HIV population group (3/180, 1.7%). Molecular screening for *Babesia* spp. resulted negative in all patients. Concerning the 3 positive patients (Table 2), 2 of them were women, who lived in the province of Bolzano and Trento, respectively. However, both of them were born in a Sub-Saharan African country, namely Mali and Nigeria. On the other hand, the positive male lived in the province of Trento and was born in Italy. None of the three patients reported history of splenectomy or transfusion. The patient who was born in Mali, had visited her country of origin in the previous 12 months. The other two patients did not report travelling outside Italy in the previous 12 months. None of the three positive patients reported any history of tick bite, nor previous blood transfusions. Moreover, only 1 patient reported outdoor activities for hobbies or professional activities. The three patients were aged between 27 and 67 years old.

**Table 1:** Demographic and clinical characteristics of enrolled patients, reported risk factors and screening results.

	PLHIV	Renal transplant	Rheumatological	Total
Number of patients	180	123	130	433
DEMOGRAPHIC CHARACTERISTICS				
N. of males (%)	140/180 (77.8%)	75/123 (70.0%)	79/130 (60.8%)	294/433 (67.1%)
Median Age	52	53	45	51
RISK FACTORS				
N. of splenectomised patients (%)	2/159 (1.3%)	0 (0.0%)	0 (0.0%)	2/179 (1.1%)
History of travelling outside Italy	27/173 (15.6%)	5/43 (11.6%)	31/129 (24.0%)	63/345 (18.3%)
History of transfusions	29/172 (16.8%)	16/40 (40.0%)	16/120 (13.3%)	61/332 (18.4%)
History of tick bite	36/172 (20.9%)	6/43 (14.0%)	19/123 (15.5%)	61/338 (18.0%)
History of TBD	0	0	0	0
History of outdoor activities at risk	79/173 (46.7%)	24/43 (55.8%)	76/124 (61.3%)	179/340 (52.6%)
History of suggestive symptoms	36/175 (20.6%)	16/43 (37.2%)	25/130 (19.2%)	77/348 (22.1%)
SCREENING RESULTS				
N. of positive serology (%)	3/180 (1.7%)	0	0	3/433 (0.7%)
N. of positive PCR	0	0	0	0

PLHIV = people living with HIV; TBD = tick-borne disease.

**Table 2:** Characteristics of the patients with positive serology for *Babesia* spp.

Patient	Sex at birth	Age	State of birth	Travelling outside Italy	History of splenectomy	History of blood transfusion	History of tick bite/TBD	Frequent outdoor activities	Suggestive symptoms
1	Female	27	Mali	Yes (Mali)	No	No	No	No	No
2	Male	67	Italy	No	No	No	No	No	No
3	Female	48	Nigeria	No	No	No	No	Yes	No

TBD = tick-borne disease.

## 4 Discussion

European studies on seroprevalence of *Babesia* spp. among the general population suggest that this protozoan infection could be more frequent than believed (Lempereur et al., 2015; Żukiewicz-Sobczak et al., 2014; Svensson et al., 2019). This could be partly explained by the frequent asymptomatic course of infection in non-immunocompromised patients, which is therefore under-recognized. Nevertheless, asymptomatic healthy individuals can be active carrier of the parasite, with a high risk of transmitting the disease through blood transfusion for several months after infection, thus leading to a silent perpetuation of the infection (Westblade et al., 2017; Popovsky, 1991). The problem could be considerable as screening for babesiosis in blood donors is not provided in Europe and immunocompromised individuals, who are at high risk of developing severe babesiosis, represent a relevant group among patients who frequently need blood transfusions. In this perspective, it is important to note that the first reported case of *B. microti* infection in Europe was transmitted through blood transfusion to a 42 year-old female with acute myeloid leukemia; a serosurvey conducted in Germany, indeed, showed that 1.7% of blood donors tested positive for *Babesia* spp. (*B. microti* or *B. divergens*) antibodies (Hunfeld et al., 2002; Hildebrandt et al., 2007).

Although severe babesiosis most frequently involves splenectomised patients (Tahir et al., 2020), other types of immunosuppression are known to be risk factors for severe *Babesia* infection. However, only a few cases of *B. divergens* and relatively more cases of *B. microti* infections have been described in normo-splenic immunocompromised patients in Europe (Hildebrandt et al., 2007; Paleau et al., 2020; Martinot et al., 2011; Gonzalez et al., 2014; Asensi et al., 2018; Chan et al., 2021; Welc-Falęciak et al., 2015; Moniuszko-Malinowska et al., 2016; Arsuaga et al., 2016; Jadin and Giroud, 1981), therefore little is known about the clinical evolution of the disease in specific groups of immunocompromised, non-splenectomised patients. Severe forms of babesiosis can also occur in immunocompetent subjects, and moderate to severe infection caused by *B. microti* or *B. divergens* have been reported in patients without any history of immunologic disorders in Europe (Martinot et al., 2011). Of note, delay in the diagnosis of this parasitic infection appears to be frequent even in the case of severe disease (Paleau et al., 2020), suggesting that misdiagnosis is not only due to the likely asymptomatic course of the infection, but also to a lack of awareness among European clinicians.

While several studies aimed to understand the ecology of zoonotic babesiosis in animals and vectors have been conducted in Italy, data including human serosurveys and/or molecular studies are lacking (Table 3). Pietrobelli et al. reported reactivity of 3/30 human sera to *B. microti* antigens in Central and Northern Italy in 2007 (Pietrobelli et al., 2007). Afterwards, Gabrielli et al. conducted a wider seroprevalence study on humans in the same areas. An overall seroreactivity of 18.7% against piroplasms (including *B. divergens*, *B. microti*, *Babesia bovis*, *Babesia canis* and *Theileria equi*) was reported, with high exposition to *B. microti* (4.6%), *B. bovis* (4.3%) and *B. divergens* (3.9%). Serological studies including testing for *B. venatorum* antibodies were never conducted in Italy, although data on animals, ticks and the human cases suggest that the seroprevalence of this pathogen should be explored in humans. To our knowledge, although the first ever reported case of *B. venatorum* human infection in the world has been described in Italy about 20 years ago (Herwaldt et al., 2003; Piccaluga et al., 2004), no more cases of human babesiosis were reported afterwards in this country. Five more cases of *B. venatorum* human infection have been reported in other European countries, 2 of which were reported in Austria. This country shares climatic and ecological features with the close Italian Northern regions, thus suggesting that *B. venatorum* could find feasible environmental characteristics for its diffusion in these areas (Häselbarth et al., 2007; Herwaldt et al., 2003; Hildebrandt et al., 2021; Blum et al., 2011; Bläckberg et al., 2018).

**Table 3:** Seroprevalence and molecular studies on human babesiosis in Italy.

Type of study	Identified pathogens/specific antigens	N. of positive samples (%)	Area	Reference
Case-report	*Babesia* EU1 (*B. venatorum*)	Single case	S. Orsola-Malpighi Hospital, University of Bologna, North-Eastern Italy	*Babesia infection in Italy, Piccaluga* et al.*; Molecular Characterization of a Non–Babesia divergens Organism Causing Zoonotic Babesiosis in Europe,* Herwaldt et al. (2003) / *Babesia infection in Italy,* Piccaluga et al. (2004)
Seroprevalence study	*B. microti*	3/488 (0.6%)	Regions of Latium, Tuscany, Umbria (Central Italy) and Venezie (North-Eastern Italy)	*Animal babesiosis: an emerging zoonosis also in Italy?* Pietrobelli et al. (2007)
Seroprevalence study	*Babesia/Theileria* *B. microti* *B. divergens*	81/423 (18.7%) 20/423 (4.6%) 17/423 (3.9%)	Northern and Central Italy	*Human exposure to piroplasms in Central and Northern Italy,* Gabrielli et al. (2014)

Our results support, in accordance with previous findings, that risk of exposure to *Babesia* spp. is not negligible in Italy, with specific concern for the North-Eastern part of the country, in accordance with other studies conducted on ticks, reservoir hosts and humans. Indeed, presence of *B. divergens* and *B. venatorum* in cattle and wild ungulates has been well described mostly in North-Eastern and Central Italy (Pietrobelli et al., 2007; Tampieri et al., n.d.; Zanet et al., 2014; Torina and Caracappa, 2007). Parallelly, several studies confirmed the diffusion of *B. microti*, *B. venatorum* and *B. divergens,* in *I. ricinus* ticks from Central and Northern Italy (Torina and Caracappa, 2007; Cassini et al., 2010; Castro et al., 2015).

The observed seroprevalences for *Babesia* infection in the provinces of Trento and Bolzano (3.6% and 3.1%, respectively) are comparable to those described by Gabrielli et al. for subjects from Central and Northern Italy, with no history of professional exposition (professional exposure in the study was meant for subjects that were persistently exposed to tick bites because of their jobs, such as forester employees, livestock keepers, veterinary practitioners, farmers and hunters) (Gabrielli et al., 2014).

Seroprevalence studies in Europe report seroprevalences ranging from 2% to 23%, with our findings being similar to those observed in Germany, but higher than those reported in Switzerland (Lempereur et al., 2015; Foppa et al., 2002; Hunfeld et al., 2002; Żukiewicz-Sobczak et al., 2014; Granström, 1997). However, comparable seroprevalences with other European countries are in contrast with the number of human cases reported in Italy, suggesting lack of awareness and likely underdiagnosis in our country. On the other hand, *I. ricinus* ticks are more dispersed in cold and moist environments and presence of zoonotic *Babesia* species is strictly connected to the presence of their main vector. Therefore, *Babesia* could be more spread in Central and Northern Europe than in the warmer Mediterranean areas such as Italy, and particularly its Southern regions (Foppa et al., 2002). In this perspective, Northern Italian regions could represent the ones that are more at risk for diffusion of zoonotic babesiosis (Ostfeld and Brunner, 2015).

In our study, all patients that tested positive to *Babesia* spp. belonged to the PLHIV group (3/180, 1.7%). In two studies conducted in Polonia and Ukraine, respectively, people infected by HIV showed higher seroprevalence for *Babesia* species than healthy blood donors, therefore appearing to be significantly more exposed (Pawełczyk et al., 2019; Bondarenko et al., 2021). On the other hand, blood donor recruitment practices and eligibility criteria for blood donations may bias the donor sample toward lower exposure risk individuals (Stone et al., 2022). Although only a few cases of human babesiosis in PLHIV are reported in the literature, the disease seems to cause severe evolution in this patient's group, requiring prolonged therapy and exhibiting high risk of recurrent or persistent parasitemia despite standard treatment (Froberg et al., 2004; Vyas et al., 2007; Mayer et al., 2007). Considering this clinical scenario, a better understanding of the potential higher susceptibility to *Babesia* spp. in PLHIV would need further investigation.

No cases of positive serology for *Babesia* spp. were reported in the rheumatologic group nor in the renal transplant group. Cases of human babesiosis in renal transplant patients have been rarely reported. They frequently present with severe evolution and need prolonged treatment, that can be longer than 6 weeks (Ather et al., 2017; Gupta et al., 1995). On the other hand, even if immunodepression has been described as a relevant risk factor for symptomatic and severe babesiosis, often associated to prolonged disease and consequently protracted treatments, there are no specific studies on the populations affected by rheumatologic diseases and undergoing immunosuppressive therapies, as performed in our study (Häselbarth et al., 2007; Hildebrandt et al., 2007; Krause et al., 2008). In a recent review on European cases of human babesiosis, no cases were described in patients receiving immunosuppressive therapies due to rheumatological diseases (Hildebrandt et al., 2021). Considering that immunosuppressing conditions are constantly growing in frequency, more information is needed about clinical and epidemiological characteristics of human babesiosis in specific groups of the immunocompromised population (Hunfeld et al., 2008).

Concerning our serologically positive patients, none of them reported any history of babesiosis-related symptoms at the time of enrollment, suggesting that, as reported in other studies (Martinot et al., 2011), *Babesia* infection can be asymptomatic or manifest with non-specific symptoms also in immunosuppressed patients. It is of interest to note that seroreactivity against *Babesia* spp. has been reported to decline starting from one year after infection and that tests frequently report only mild or doubtful seropositivity, with the high probability of turning negative in a short time (Martinot et al., 2011; Piccaluga et al., 2004; Bloch et al., 2016). This could also suggest the possibility of underestimation of exposure to *Babesia* spp. in serological surveys, especially in the case of infections contracted a long time before testing.

A limit of this study is that serological assays were based on *B. divergens* antigens; since the specificity for *B. divergens* of the utilized *home-made* serological assay is not established, it is not possible to determine whether seropositivity against other *Babesia* species could have passed undetected in this work. Furthermore, little is known about sensitivity of serological tests for *Babesia* spp. in immunocompromised patients, with the risk of even greater underestimation in this group of patients, that could present with false-negative results due to immune disfunction. For this reason, and with the aim to detect recent infections that could have not undergone seroconversion yet, molecular tests were also performed as a screening test in our study. However, no positive results were recorded among seronegative patients, nor in any of the 3 seropositive patients.

Similar to previous studies (Lempereur et al., 2015), our *Babesia*-positive patients were in their middle age (medium age 47 years), confirming that, although older age is a risk factor for severe forms of babesiosis, elderly population is less exposed to the pathogen due to less frequent outside activities (Lempereur et al., 2015). Concerning the other risk factors, only one among the positive patients reported frequent outdoor activities and none reported history of splenectomy, transfusion, tick-bite or tick-borne disease, although all patients live in areas were presence of *I. ricinus* ticks is among the highest in Italy and tick-bites could have passed unnoticed.

Two out of the 3 serological positivities for *Babesia* spp. were detected in patients who were born in Sub-Saharan Africa, while the third patient was born in Italy and did not report history of travelling abroad. Therefore, an autochthonous infection caused by *Babesia* spp. is assured only for the latter. As regarding diffusion of babesiosis in the African continent, presence of *Babesia* spp. has been reported in animals and human infections have only been hypothesized, with no clear data about species identification. One case was confirmed to be caused by *B. microti* in Equatorial Guinea, but in that case importation of the infection from Spain could not be ruled out (Arsuaga et al., 2018). Only a few studies on human babesiosis have been conducted in the African continent (Bush et al., 1990; El-Bahnasawy et al., 2011; Gabrielli et al., 2016b; Bloch et al., 2018; Ayeh-Kumi et al., 2022). A survey conducted in the Democratic Republic of Congo enabled the molecular identification of *B.microti* in 6.2% out of 306 enrolled children (Gabrielli et al., 2016b), suggesting that diffusion of human babesiosis could be higher than previously expected and often misdiagnosed as malaria, due to similar clinical and morphological features. Moreover, cross-reactivity between *Plasmodium* spp. and *Babesia* spp. antibodies has been hypothesized (Bloch et al., 2018). This hypothesis could not be ruled out for the two *Babesia*-positive patients in our study group, who originated from countries that are endemic for malaria, although the clusterisation of the three positive cases in one specific area of the study could suggest a correlation with local epidemiology rather than a cross-reaction. More studies are needed to understand *Babesia* spp. distribution in the African continent and to better discriminate whether our patients could have been infected in their countries of origin (Arsuaga et al., 2018; Bloch et al., 2018; Ayeh-Kumi et al., 2022).

## 5 Conclusions

In conclusion, more information is needed to understand the epidemiology and distribution of *Babesia* spp. in Italy at the animal reservoir, vector and human interface and the risk for human exposure to *Babesia* spp. should be further characterized. Understanding the real clinical burden of babesiosis in Italy, as well as estimating the spread of the infection among asymptomatic individuals, appears to be crucial in order to establish the risk to which immunocompromised patients are exposed. *Babesia* spp. ecology is destined to change, in connection with climate change, environmental modifications and variations in human activities; to understand this dynamic process, increased knowledge on human babesiosis is needed, which should be analyzed in a “One-Health” perspective.

## Acknowledgments and funding

The study was financed by the Italian Ministry of Health, in the context of a research program (Ricerca Finalizzata 2016, project code RF-2016-02361931). IRCCS Sacro Cuore-Don Calabria Hospital was also funded by Italian Ministry of Health (Fondi Ricerca Corrente - Linea 2 Progetto 7).

## CRediT authorship contribution statement

**Anna Barbiero:** Writing – original draft, Methodology, Investigation, Formal analysis, Data curation. **Simona Gabrielli:** Writing – review & editing, Investigation, Formal analysis, Data curation. **Lapo Dani:** Writing – original draft, Investigation, Data curation. **Michele Spinicci:** Writing – review & editing, Investigation, Data curation. **Filippo Lagi:** Writing – review & editing, Supervision, Methodology, Investigation, Data curation. **Gregorio Basile:** Investigation, Data curation. **Francesca Nacci:** Investigation, Data curation. **Antonia Mantella:** Investigation, Data curation. **Seble Tekle Kiros:** Investigation, Data curation. **Angela Pieri:** Writing – review & editing, Supervision, Investigation, Data curation. **Andrea Delama:** Writing – review & editing, Supervision, Investigation, Data curation. **Chiara Piubelli:** Writing – review & editing, Investigation, Data curation. **Salvatore Scarso:** Writing – review & editing, Investigation, Data curation. **Andrea Angheben:** Writing – review & editing, Supervision, Investigation, Data curation. **Marcello Feasi:** Writing – review & editing, Supervision, Investigation, Data curation. **Bianca Granozzi:** Writing – review & editing, Investigation, Data curation. **Giorgia Comai:** Writing – review & editing, Investigation, Data curation. **Stefania Varani:** Writing – review & editing, Supervision, Resources, Project administration, Methodology, Investigation, Funding acquisition, Formal analysis, Data curation, Conceptualization. **Lorenzo Zammarchi:** Writing – review & editing, Supervision, Methodology, Investigation, Conceptualization. **Alessandro Bartoloni:** Writing – review & editing, Supervision, Project administration, Investigation, Formal analysis, Data curation, Conceptualization.

## Declaration of competing interest

The authors report there are no competing interests to declare.
